# Visceral Adipose MicroRNA 223 Is Upregulated in Human and Murine Obesity and Modulates the Inflammatory Phenotype of Macrophages

**DOI:** 10.1371/journal.pone.0165962

**Published:** 2016-11-03

**Authors:** Jeffrey A. Deiuliis, Rafay Syed, Dheeraj Duggineni, Jessica Rutsky, Palanivel Rengasamy, Jie Zhang, Kun Huang, Bradley Needleman, Dean Mikami, Kyle Perry, Jeffrey Hazey, Sanjay Rajagopalan

**Affiliations:** 1 Department of Medicine, Division of Cardiovascular Medicine, University of Maryland, Baltimore, Baltimore, MD 21201, United States of America; 2 Dorothy Davis Heart and Lung Institute, The Ohio State University College of Medicine, Columbus, OH 43210, United States of America; 3 Department of Biomedical Informatics, The Ohio State University College of Medicine, Columbus, OH 43210, United States of America; 4 Department of Surgery, The Ohio State University College of Medicine, Columbus, OH 43210, United States of America; Brown University Warren Alpert Medical School, UNITED STATES

## Abstract

Obesity in humans and mice is typified by an activated macrophage phenotype in the visceral adipose tissue (VAT) leading to increased macrophage-mediated inflammation. microRNAs (miRNAs) play an important role in regulating inflammatory pathways in macrophages, and in this study we compared miRNA expression in the VAT of insulin resistant morbidly obese humans to a non-obese cohort with normal glucose tolerance. miR-223-3p was found to be significantly upregulated in the whole omental tissue RNA of 12 human subjects, as were 8 additional miRNAs. We then confirmed that miR-223 upregulation was specific to the stromal vascular cells of human VAT, and found that miR-223 levels were unchanged in adipocytes and circulating monocytes of the non-obese and obese. miR-223 ablation increased basal / unstimulated TLR4 and STAT3 expression and LPS-stimulated TLR4, STAT3, and NOS2 expression in primary macrophages. Conversely, miR-223 mimics decreased TLR4 expression in primary macrophage, at the same time it negatively regulated FBXW7 expression, a well described suppressor of Toll-like receptor 4 (TLR4) signaling. We concluded that the abundance of miR-223 in macrophages significantly modulates macrophage phenotype / activation state and response to stimuli via effects on the TLR4/FBXW7 axis.

## Introduction

Visceral adipose tissue (VAT) in obese humans, particularly those with insulin resistance, is typified by the activation of resident and infiltrating inflammatory cells, both innate and adaptive in nature [1, 2]. Concomitant with this pro-inflammatory shift in the VAT is a reduction in immunosuppressive cells, such as T regulatory cells [3]. The severity of metabolic disease in humans and mice correlates well with various markers of inflammation, including macrophage activation; however, the mechanisms regulating VAT inflammation are of particular interest for multiple reasons, including the effect of VAT-derived cytokines on muscle insulin resistance [4]. This study aimed to identify miRNAs likely involved in obesity-related VAT inflammation in humans, in the hopes that a better understanding of meta-flammation associated miRNAs may provide therapeutic targets for modulating macrophage-mediated inflammation. As of late, technological advances using small-oligos in human therapeutics have matured rapidly, especially anti-miRNA approaches. Our study suggests that a miR-223-FBXW7-TLR4 axis plays a strong role in the macrophage inflammatory phenotype, with ablation of miR-223 regulating multiple pathways including Toll-like receptor 4 (TLR4), STAT3, and inducible nitric oxide synthase (NOS2) signaling. TLR4 signaling is known to be involved in the etiology of meta-flammation and LPS stimulation of TLR4 is known to downregulate miR-223 expression during macrophage polarization [5]. Recent studies have shown a role for FBXW7 in attenuating TLR4 expression [6] and, thereby, macrophage response to lipopolysaccharide and other ligands such as palmitic acid which is elevated in obesity and insulin resistance. We hypothesized a link between macrophage miR-223 expression and TLR4 signaling-mediated changes in FBXW7 expression, which have not been previously reported.

## Methods

### Human Participants

This study recruited and obtained visceral adipose samples from lean and morbidly obese surgical patients (lean, BMI<30; obese, BMI≥40). Two patient cohorts were examined: cohort 1 (n = 6/group; lean—4 female, 2 male, obese—5 female, 1 male), cohort 2 (n = 19/group; lean– 9 female, 10 male; obese—11 female, 8 male). Samples were obtained from the greater omentum at the time of endoscopic hernia repair (lean subjects) and gastric bypass (bariatric) surgery (obese subjects) between the years 2011 and 2013. The Office of Responsible Research Practices, Human Institutional Review Board (IRB) of the Ohio State University (protocol 2008H0177) and the University of Maryland, Baltimore IRB (HP-00058301), approved this study and its procedures. Informed consent was obtained in writing.

### Animals

Mice were obtained and handled according to a UMB, IACUC protocol #0514008. Animals were euthanized with carbon dioxide and tissues harvested immediately and snap frozen for further analysis.

### Blood values

Fasting blood was drawn the morning of surgery by venipuncture. Blood chemistry values, measured by the Department of Clinical Laboratories at The Ohio State University Medical Center, are provided in the results section. Methodologies for each assay can be provided upon request. HOMA-IR was calculated using the following equation: [glucose (mg/dl) x insulin (mU/L)/405]. Values are in mg/dl unless otherwise noted in table legend.

### RNA Isolation and Exiqon microRNA Array

Whole adipose tissue was homogenized in TRIzol^®^ reagent (Life Technologies), followed by lipid removal. RNA quantity and quality were determined by spectrophotometry (nanodrop) and by Agilent Bioanalyzer RNA assays. All RNA samples had RNA integrity numbers (RIN) above 7. 1 μg of RNA was hybridized to an Exiqon miRCURY^™^ LNA Array 5th generation (product number 208300-A (208301-A / 208302-A, slide batch 33011) using miRBase 15.0 + miRPlus. Analysis of the scanned slides showed that the labeling was successful as all capture probes for the control spike-in oligo nucleotides produced signals in the expected range. The quantified signals (background corrected) were normalized using the global Lowess (LOcally WEighted Scatterplot Smoothing) regression algorithm. In the expression matrix, all capture probes with both Hy3 (sample) and Hy5 (common reference pool) signals lower than 1.5x of the median signal intensity of the given slide were excluded from analysis. Only log2(Hy3/Hy5) ratios which passed the filtering criteria on variation across samples were used. Student’s t-test was performed between lean and obese patient groups. The miRNAs with mean expression level change above 1.3 fold, and p value < 0.05 were correlated with patient phenotypic traits (BMI, CRP level, fasting insulin level, and HOMA index). Pearson correlation coefficient and the corresponding values were obtained using Matlab corr function.

### Taqman Probed-Based qPCR measure of microRNA expression

TaqMan^®^ MicroRNA Assays by ABI were used to verify miRNA expression according to manufacturer’s instructions. Briefly, miRNA cDNA was synthesized using RT primers provided by ABI. RNU44/SNORD44, small nucleolar RNA, C/D box 44, (NR_002750) was used as an endogenous control for calculation of relative expression between samples. Reactions used ABI Taqman master mix with probe provided by ABI in a Roche LightCycler 480.

### SYBR green-based qPCR

Quantitative PCR was performed as published previously [7]. Primers for F-box and WD-40 domain protein 7 (FBXW7) isoform measurement were based on the following publications [8, 9]. FBXW7 pan primer; 5’- CGAGACTTCATCTCCTTGCTTCC-3’ (for), 5’-CCAGAGAAGGTTATCCTCAGCC-3’ (rev).

### hFBXW7 3’-UTR Luciferase Reporter Assay

The 3’ UTR was cloned into a TOPO TA cloning vector (Life Technologies, Grand Island, NY) with primers that introduced SpeI/SacI restriction enzyme sequences to the hFBXW7-3’-UTR amplicon. TOPO-cloning of the hFBXW7 3’-UTR was validated by sequencing before restriction enzyme digestion and purification of the cloned fragment. The SpeI/SacI hFBXW7-3’UTR digestion fragment was ligated to the prepared pMIR-REPORT^™^ miRNA Expression Reporter Vector System (Life Technologies, Grand Island, NY) according to manufacturer’s instructions. pMIR-REPORT-hFBXW7 construct was validated by sequencing. The pMIR-REPORT-hFBXW7-WT construct was mutated at 4 miR-223 seed / binding sites using the Stratagene Quikchange Lightning Multi Site-Directed Mutagenesis Kit (Agilent, Santa Clara, CA) according to manufacturer instructions. Colonies were screened for quadruple miR-223 mutations and validated by sequencing. 10^6^ HeLa cells (ATCC) were co-transfected with pMIR-REPORT-hFBXW7-WT or pMIR-REPORT-hFBXW7-quadruple mutant (MUT), miR-223 mimic—50nM (*mir*Vana^™^ by Life Technologies), and β-gal transfection control plasmid using an Eppendorf Multiporator^™^. Cells were recovered and grown for 36 hours in phenol-red free DMEM (4.5 g/L D-glucose) with 20% FBS followed by cell lysis for luciferase and β-gal quantification using the Dual-light^™^ System (Life Technologies) according to manufacturer instructions with luminescence measured on a Berthold XS^3^ LB 960 luminometer using plate injection. The experiment was performed three times.

### Primary mouse macrophage culture and treatment

WT and miR-223^-/-^ mice were obtained from Jackson Laboratories. Primary Bone Marrow Derived Macrophages (BMMs) were cultured as previously described,^1^ except bones were flushed with Bone Marrow Media (DMEM, 20% L-cell conditioned media, 10% FBS, 1% Pen/Strep), media was renewed every other day, and bone marrow cells were differentiated for 7 days before use. BMDMs were seeded in bone marrow media at appropriate densities for assays and given 24 hours to equilibrate before treatment. Treatments consisted of unstimulated controls, LPS (100 ng/ml, LCD25; List Biological Laboratories) or a combination of LPS (100 ng/mL) & IFNγ (100 ng/ml) for 24 hours.

### Primary mouse macrophage transfection with miR-223 mimic and oligo control

miR223KO BMMs were grown until confluent (day 7), scraped in warm 1X PBS, before the appropriate number of cells were aliquoted, spun and resuspended in Eppendorf hypo-osmotic buffer at 10^6^ cells/ml. After 10 minutes in the hypo-osmotic buffer, cells were electroporated (Eppendorf Multiporator^™^) at 1200V, 50μs in the presence of 500nM of miR-223 mimic or mimic control oligo. BMMS were plated in recovery media (20% FBS, phenol red free with no antibiotics) overnight, after which media was changed to growth media containing 100 ng/ml LPS. BMMs were harvested for RNA 36 hours after LPS stimulation.

### Immunoblotting

Cells were washed 2x with sterile PBS and lysed at approximately 500,000 cells/mL of ice-cold lysis buffer (80mM Tris-HCL pH 6.8, 15% glycerol, 2% SDS and 10 uL/mL commercial protease/phosphatase inhibitor cocktail). Cell lysis was followed by 1 minute of sonication and then centrifugation at 17,500rpm for 15 minutes. The protein content of cell lysate was determined using a bicinchoninic acid (BCA) protein assay kit (Pierce Chemical, Rockford, IL). Proteins were separated by SDS–PAGE using a mini-Protean system (Bio-Rad, Hercules, CA) and wet-transferred to a PVDF membrane (Amersham Biosciences, Uppsala, Sweden), followed by blocking with 3% BSA in 1x-TBST (0.1% Tween-20) overnight. Membranes were incubated with primary antibody (in 3% BSA) specific to iNOS & FBXW7 (Abcam; 1:1000, 1 hr at RT); p-STAT3 & STAT3 (Santa Cruz Biotechnology; 1:500, overnight at 4°C); TLR4 (Novus Biologicals NB100-56580; 1:1,000, 2hr at RT) and β**-**actin (Cell Signaling Technologies; 1:3000, 1 hr at RT). After washing with 1X TBST, blots were incubated with the appropriate HRP-conjugated secondary antibody for 1 hr at room temperature (RT). Blots were washed before addition of ECL plus^™^ (Amersham Biosciences) and detection of bands with Kodak Biomax^™^ film.

### Data Analysis

All data, with the exception of array data, were analyzed using Graphpad Prism software (Version 6), two-tailed unpaired student’s t-test or one-way ANOVA followed by Boneferroni’s post-hoc test where appropriate. All data are expressed as mean ± SEM unless otherwise specified. P value of <0.05 was considered statistically significant.

## Results

### Whole miRNome profiling in omental adipose

**Fig 1:**
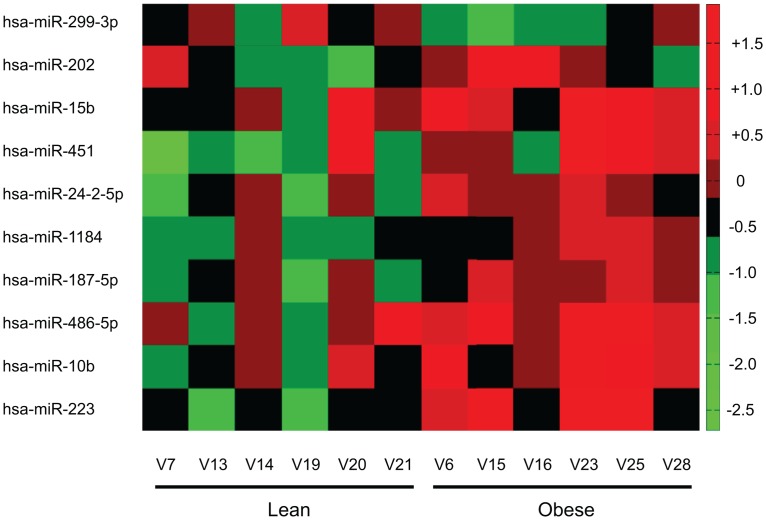
Heatmap of miRNA expression for those with a significant difference between groups. The heatmap is organized by group (lean or obese) on x-axis and increasing significance between groups from top to bottom on the y-axis. Red indicates an increase and green/black a decreased relative expression per sample (n = 6 per group).

Patient samples were selected for significantly disparate BMIs (27±0.6 lean vs. 50±3 obese) and HOMA-IR (cut off values: lean <0.6 and obese >3), but similar age (41.7±6 lean vs 47.6±5 obese; NS), sex, and blood lipid profiles (Table 1). 12 microRNAs passed the threshold of mean fold change > 1.3 and p-value < 0.05 between the lean and obese groups, with one microRNA significantly downregulated (miR-299-3p) and nine significantly upregulated (miR-202-3p, -15b-5p, -451, -24-2-5p, 1184, -187-5p, -486-5p, -10b-5p, and -223-3p). Normalized miRNA expression is depicted in a heatmap (Fig 1A). In order to focus our investigations on a particular miRNA / molecular pathway, we performed Affymetrix GeneChip analysis on the same RNA from patients in cohort 1 and used Qiagen Ingenuity Pathway Analysis software to overlay Exiqon miRNA array expression with Affymetrix gene expression of *predicted* miRNA-gene targets. All gene expression data was used in initial analyses followed by subsequent filtering for mean fold-change greater or equal to 1.2 in target mRNA between lean and obese groups. However, gene expression changes between groups did not reach statistically significance likely due to sample size limitations.

**Table 1:** Cohort 1—Patient Characteristics—MicroRNA Arrays.

	Lean	Obese
Age	41.7±6	47.6±5
Gender	4F; 2M	5F; 1M
BMI	27±0.6	50±3*
Glucose	90±10	143±20*
Insulin	3.4±1.2	19.3±3.1*
HOMA-IR	0.77±0.3	6.7±1.3*
QUICKI	0.43±0.02	0.29±0.01*
HbA1c (%)	5.6±0.1	6.6±1
HbA1c^†^	38±1.1	49±11
TG	123±31	144±32
CRP	2.4±0.8	8.3±3.4
TC	171±9	168±14
HDL	44±4.3	41±7.5
LDL	102±11	98±10
TZD	0	3

All values are mean ± SEM where applicable

* indicates p<0.05 via student t-test

^†^ indicates (mmol/mol); CRP (c-reactive protein), glucose, and lipid panel constituents are in mg/dl; Insulin is μIU/ml; TZD, count of patients using thiazolidinedione drugs; TC, total cholesterol; TG, fasting triglycerides.

### Pearson correlations of top 10 miRNAs and clinical parameters of inflammation and insulin resistance

Pearson correlations coefficients between the 10 differentially expressed miRNAs with 4 widely used clinical parameters: BMI, fasting insulin, HOMA-IR, and CRP. miR-486-5p, -15b and -451 were most significantly (p<0.05) correlated with the measures (Table 2). miR-15b was recently shown to be upregulated in the plasma of obese humans and suggested as a marker for insulin resistance [10]. miR-10b was the most highly correlated miRNA with BMI, but also correlated strongly to fasting insulin and HOMA measurements. miR-223 was the second most correlated with BMI. miR-187, miR-202, and miR-299-3p were not significantly correlated with any of the four markers. Based on the limitations of array methods to quantify miRNA expression and lack of cellular specificity of whole adipose RNA, we pursued Taqman probe-based quantification of candidate miRNA levels in fractionated adipose RNA in a second, larger confirmation cohort of patients. We aimed to validate differential miRNA expression in a larger population and to gain insights into adipose tissue fraction-specific miRNA expression.

**Table 2:** Pearson correlation coefficients—miRNA expression and patient parameters.

miRNA	BMI	Fasting Insulin	HOMA	CRP
miR-223	0.80 p = 0.002	0.66 p = 0.027	NS	0.63 p = 0.038
miR-10b	0.83 p = 8.65E-4	0.68 p = 0.021	0.70 p = 0.016	NS
miR-486-5p	0.74 p = 0.006	0.72 p = 0.013	0.70 p = 0.016	0.68 p = 0.021
miR-187-5p	NS	NS	NS	NS
miR-1184	0.73 p = 0.007	0.63 p = 0.037	0.64 p = 0.036	N/S
miR-24-2-5p	0.69 p = 0.013	0.69 p = 0.019	0.65 p = 0.030	N/S
miR-451	0.74 p = 0.006	0.61 p = 0.047	0.61 p = 0.044	0.61 p = 0.046
miR-15b	0.74 p = 0.006	0.67 p = 0.023	0.66 p = 0.028	0.62 p = 0.040
miR-202	NS	NS	NS	NS
miR-299-3p	NS	NS	NS	NS

First values in each cell are Pearson correlation coefficients “r” followed by p-value for comparison. Correlation coefficients are not given if p-values >0.05; NS, Not Significant.

### miRNA expression in a larger cohort of patients

**Fig 2:**
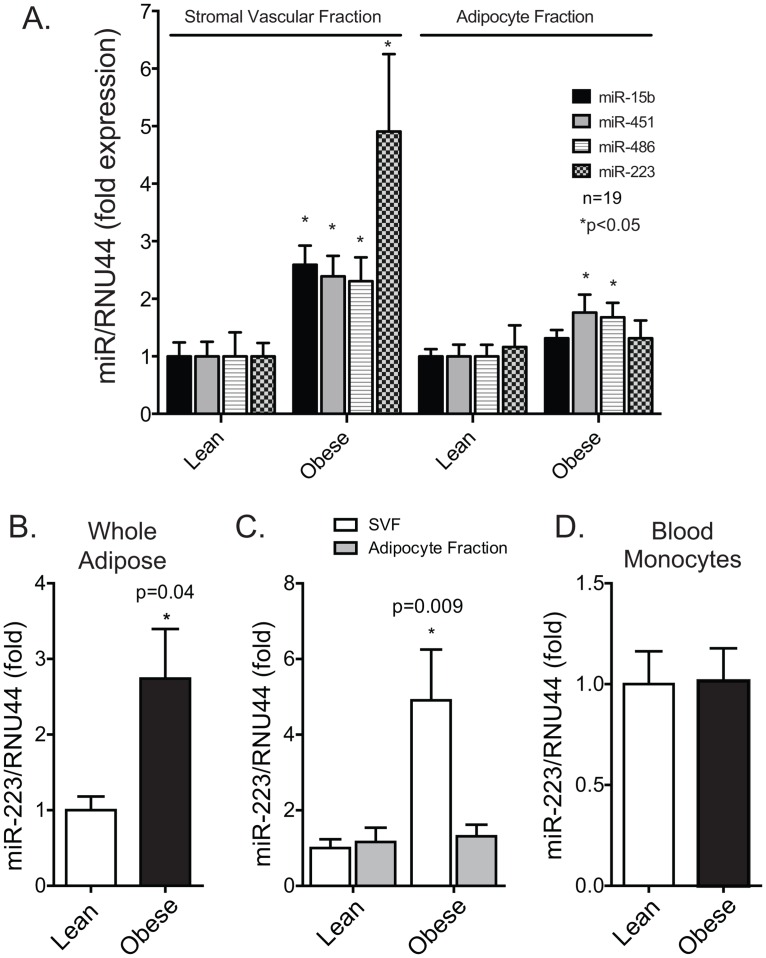
TaqMan qPCR validation of microRNAs in stromal vascular, adipocyte, and blood monocyte cells in confirmation cohort. **(**A**)** Freshly isolated human adipose was fractionated to stromal vascular and adipocyte fractions from lean and obese individuals and RNA isolated. miRNA levels were examined by Taqman probe based qPCR with data expressed as fold change relative to “housekeeping” RNA, RNU44 (human only). (B) Human miR-223 expression; hsa-miR-223 levels in whole human visceral adipose tissue (omentum) relative to RNU44, (C) hsa-miR-223 levels in the SVF and adipocyte fractions of digested human adipose/omentum relative to RNU44, and (D). hsa-miR-223 levels in circulating human blood monocytes isolated with Ficoll-Paque Plus (GE Healthcare).

The visceral adipose of a second cohort consisting of 19 patients per group (Table 3) was processed for stromal vascular (SVF) and adipocyte fractions immediately post-collection. Plasma blood mononuclear cells (PBMCs) were also collected. We found that miR-15b, -451, -486, and -223 were significantly upregulated in the SVF, with miR-223 being the most dramatically increased in the obese group (Fig 2A). Differences between groups were lower in the adipocyte fraction, with miR-451 (1.8-fold, p<0.05) and -486 (1.7-fold, p<0.05) being modestly upregulated. miR-223 was not altered in the adipocyte fraction or in PBMCs (Fig 2D). The differential expression of candidate miRNAs between groups was similar whether normalized to total RNA loaded per assay or RNU44. miR-223 was significantly upregulated in whole adipose in cohort 2 (Fig 2B).

**Table 3:** Cohort 2—Patient Characteristics.

	Lean	Obese
Age	47.1±3.5	44.9±2.4
Gender	9F; 10M	11F; 8M
BMI	25.1±0.5	51.7±1.9*
Glucose	88±1.9	138±11*
Insulin	4.5±0.7	20.1±4.2*
HOMA-IR	0.99±0.2	7.8±2.4*
QUICKI	0.40±0.01	0.31±0.01*
HbA1c (%)	5.4±0.1	6.3±0.3*
HbA1c^†^	36±1.1	45±3.3*
TG	86.2±18	133±16
CRP	2.0±0.7	8.3±1.3
TC	173±13	170±10
HDL	52±4	37±2*
LDL	104±11	106±8
TZD	0	5

The average age of individuals was similar to cohort 1. Individuals in the obese group of confirmation cohort 2 had significantly higher BMI, glucose, insulin, HOMA-IR, QUICKI, HemA1c, and HDL-C compared to cohort 2 control subjects. All values are mean ± SEM where applicable

* indicates p<0.05 via student t-test

^†^ indicates (mmol/mol)

### miR-223 targets the protein ubiquitination pathway

**Fig 3:**
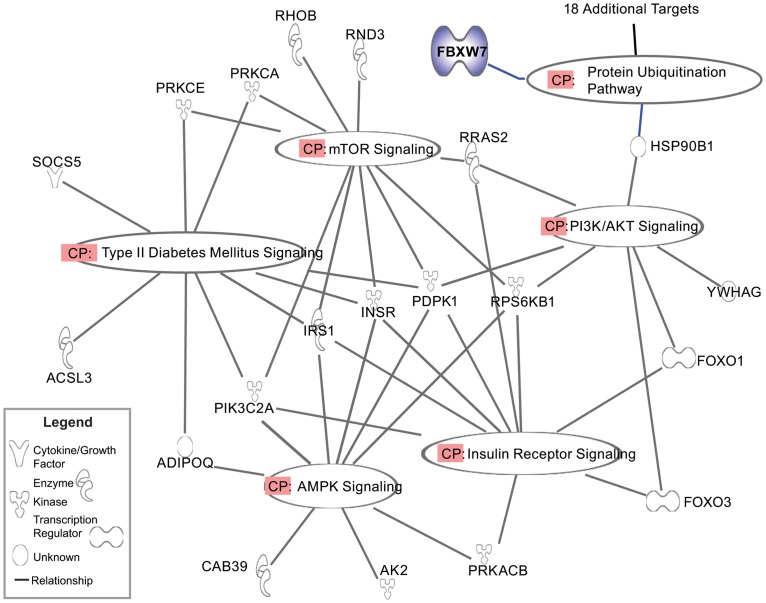
Pathway analysis of miR-223 gene targets using IPA^®^ software by Ingenuity Systems (Qiagen). KEGG Canonical pathways are labeled (CP). All targets are depicted by the official gene symbol and function of protein product can be found in legend. Genes symbols are: ADIPOQ, Adiponectin; PIK3C2A, phosphoinositide-3-kinase (PI3K), class 2, alpha polypeptide; IRS1, insulin receptor substrate 1; INSR, insulin receptor; PDPK1, 3-phosphoinositide dependent protein kinase-1; RPS6KB1, ribosomal protein S6 kinase, 70kDa, polypeptide 1; PRKACB, protein kinase A, cAMP-dependent, catalytic, beta.

In order to identify an appropriate gene target for miR-223, we utilized 11 separate tools for maximal predictive power (DIANA-microT (http://www.diana.pcbi.upenn.edu/cgi-bin/micro_t.cgi), MicroInspector (http://mirna.imbb.forth.gr/microinspector/), miRanda (http://www.microrna.org/), MirTarget2 (http://mirdb.org/), miTarget (http://cbit.snu.ac.kr/~miTarget/), NBmiRTar (http://wotan.wistar.upenn.edu/NBmiRTar/), PicTar (http://pictar.bio.nyu.edu/), PITA (http://genie.weizmann.ac.il/), RNA22 (http://cbcsrv.watson.ibm.com/rna22.html), RNAhybrid (http://bibiserv.techfak.uni-bielefeld.de/rnahybrid/), and TargetScan (http://www.targetscan.org/)). We found that FBXW7 (NCBI Gene 55294) was a highly predicted target within all platforms. FBXW7 has previously been shown to be a target of miR-223 [11], however the FBXW7-miR-223 axis in macrophage inflammation has not been reported. Ingenuity pathway analysis (IPA) was used to sort potential targets of miR-223 by canonical pathways potentially relevant to diabetes, cellular energy utilization, and inflammation (Fig 3). The most strongly enriched canonical pathway was protein ubiquitination, with 19 targets with annotation to this group. This finding further solidified our focus on FBXW7.

**Fig 4:**
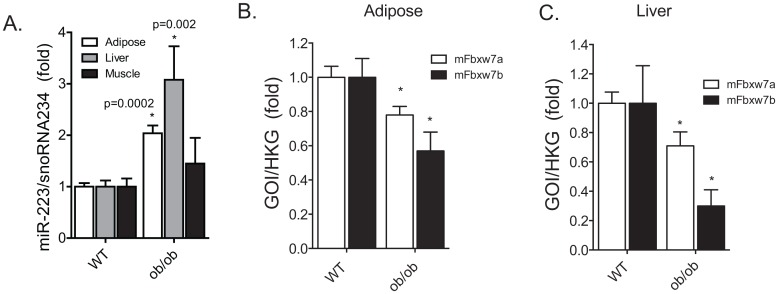
Murine obesity demonstrated increased miR-223 expression and repressed FBXW7 levels. (A) mmu-miR-223 levels were significantly increased in the whole adipose and liver tissues, but not in skeletal muscle. (B) FBXW7α and -β gene expression by qPCR in WT and *ob/ob* mice whole adipose tissue, white column—alpha isoform; black column—beta isoform. (C) FBXW7α and -β gene expression by qPCR in WT and *ob/ob* mice whole liver, white column—alpha isoform; black column—beta isoform.

FBXW7 protein is one of four subunits of an ubiquitin protein ligase complex called SKP1-cullin-F-box. FBXW7 functions in substrate recognition of the ubiquitination complex. Recently, the Sterneck group has clearly shown the role of FBXW7 in attenuating TLR4 signaling processes in macrophages [6]. Thus, we believe that the miR-223-FBXW7 axis in macrophage inflammation is an important mechanism in balancing TLR4-mediated inflammation in macrophages and is relevant to metaflammation. FBXW7 has 2 isoforms, alpha and beta. In order to assess the significance of miR-223 as a broadly conserved pathophysiologic change, we isolated RNA from the adipose, liver and muscle of *ob/ob* mice at approximately 20 weeks of age. We found that miR-223 was significantly upregulated in the liver and adipose of obese mice compared to lean wild type C57BL/6 control mice; expression was normalized to snoRNA234 in murine samples (Fig 4A). We found FBXW7α and -β were significantly decreased in both the whole adipose and whole liver (Fig 4B and 4C) of *ob/ob* mice, however, we found no significant difference in human SVF for hFBXW7α and -β (data not shown).

**Fig 5:**
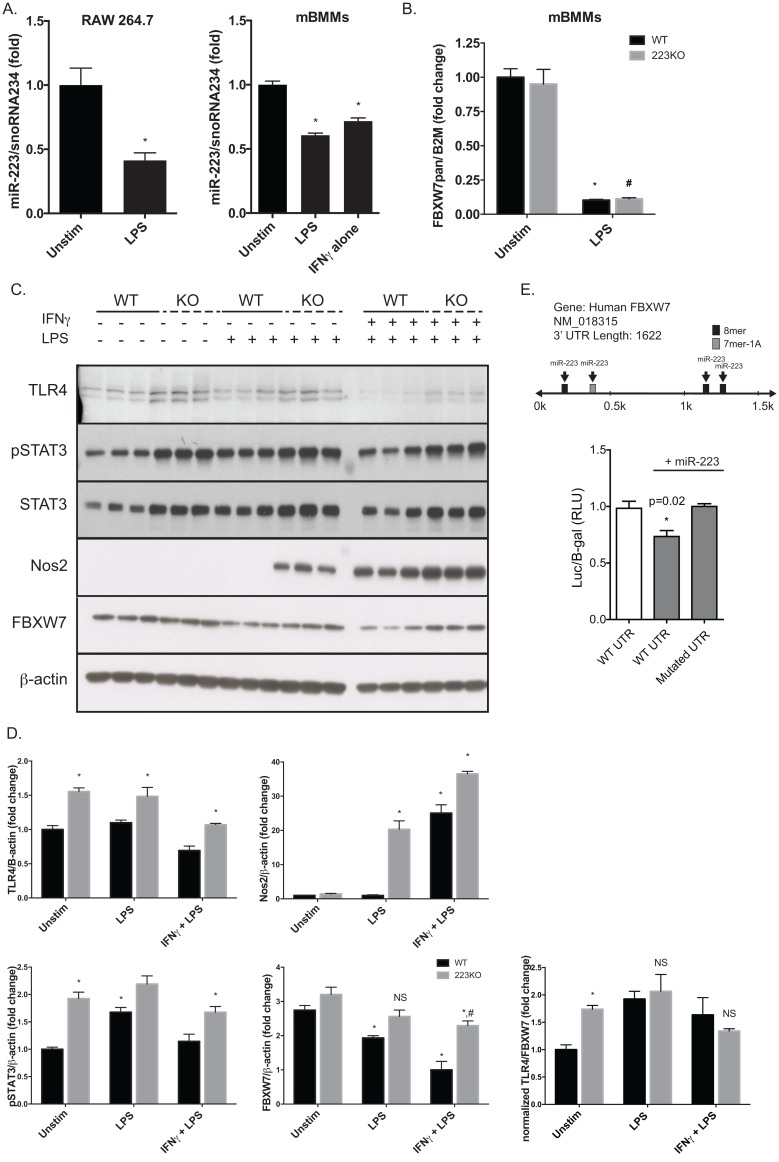
miR-223 null macrophages have enhanced inflammatory signaling and increased FBXW7 levels. (A) Effect of LPS (100ng/ml) on mmu-miR-223 levels in the mouse macrophage cell line Raw264.7 and in primary mouse macrophages from C57BL/6J mice as measured by qPCR, relative to snoRNA234. (B) LPS treatment dramatically decreases FBXW7 mRNA levels in primary mouse macrophages at 24 hours (normalized to B2M) by qPCR. (C) Immunoblot data examining response of primary macrophages from WT and miR-223 knockout mice (KO) to LPS and combined IFNγ/LPS; 50μg of whole cell lysate was loaded per lane and run on a denaturing SDS PAGE gel. Densitometry and statistics are presented in (D). (E) hFBXW7 3’-UTR luciferase reporter assay experiment. Data is expressed as luciferase-hFBXW7 3’-UTR activity over β-galactosidase activity as measured by luminescence. “+miR-223” indicates co-transfection of miR-223 mimic. “WT UTR” indicates a vector containing un-mutated hFBXW7 3’-UTR and “Mutated UTR” indicates a vector containing hFBXW7 3’-UTR that has been mutated at all four miR-223 seed sequences. All values are expressed as ± SEM; * indicates p<0.05 via student t-test.

In order to better understand the interplay between miR-223, FBXW7 and macrophage inflammatory signaling, we compared primary bone marrow derived macrophages (mBMMs) from wild type and miR-223 whole body knockout mice. IFNγ and/or LPS activation of mBMMs and RAW264.7 cells resulted in a 50% decrease in miR-223 levels (Fig 5A & 5B), indicating that TLR4 signaling regulates miR-223 transcription or pri-miRNA maturation. In agreement with the Sterneck reports, LPS dramatically decreased FBXW7 expression at the mRNA (Fig 5B) and protein levels (Fig 5C) in WT mice. miR-223 null macrophages, however, showed no significant decrease in FBXW7 protein levels with LPS treatment compared to unstimulated WT or null vehicle controls. Dual stimulation with IFNγ and LPS resulted in a significant, but modest decrease in miR-223 null macrophages, significantly contrasted by the dramatic decrease of FBXW7 in WT macrophages. pSTAT3 and total STAT3 levels in miR-223 null macrophages were clearly elevated over WT, as were levels of NOS2, indicating an enhanced inflammatory response to LPS and IFNγ/LPS. These data suggest that miR-223 plays a significant role in both pro and anti-inflammatory pathways in mature macrophages and that further elucidation of the intricacies of this regulation is important to inflammatory diseases.

Human primary macrophages are largely recalcitrant to transfection, thus for miR-223-FBXW7 interactions in human cells we utilized a luciferase reporter assay system (Fig 5E). The entire human FBXW7 3’-UTR was inserted downstream of the luciferase gene in the pmiR-REPORT vector. We found that co-transfection of a miR-223 mimic with the hFBXW7 3’-URT (WT UTR) plasmid decreased luciferase expression / function to a significant degree, demonstrating that miR-223 mimics can repress a surrogate of human FBXW7 expression. In contrast, the miR-223 mimic resulted in no change in luciferase activity when co-transfected with the hFBXW7 3’-UTR engineered with mutated miR-223 seed / binding sites, compared to control.

### miR-223 suppresses macrophage activation via inducible nitric oxide synthase

In order to characterize how abolition of miR-223 affects macrophage polarization, we measured the expression of several pro-inflammatory related proteins. In agreement with previous studies [12], we found that miR-233 null macrophages expressed significantly higher amounts of phosphorylated and total-STAT3, in unstimulated, LPS-activated or LPS/IFNγ conditions (Fig 5C). It is also well documented that pro-inflammatory macrophages produce elevated levels of nitric oxide (NO). Our data shows that miR-223 null macrophages express significantly higher levels of inducible nitric oxide synthase (NOS2) (Fig 5C). To our knowledge, this is the first study to report that miR-223 affects NOS2 levels and provides an additional mechanism by which miR-223 expression in macrophages modulates an activated phenotype.

### miR-223 mimic reduces TLR4 expression in LPS-stimulated macrophages

**Fig 6:**
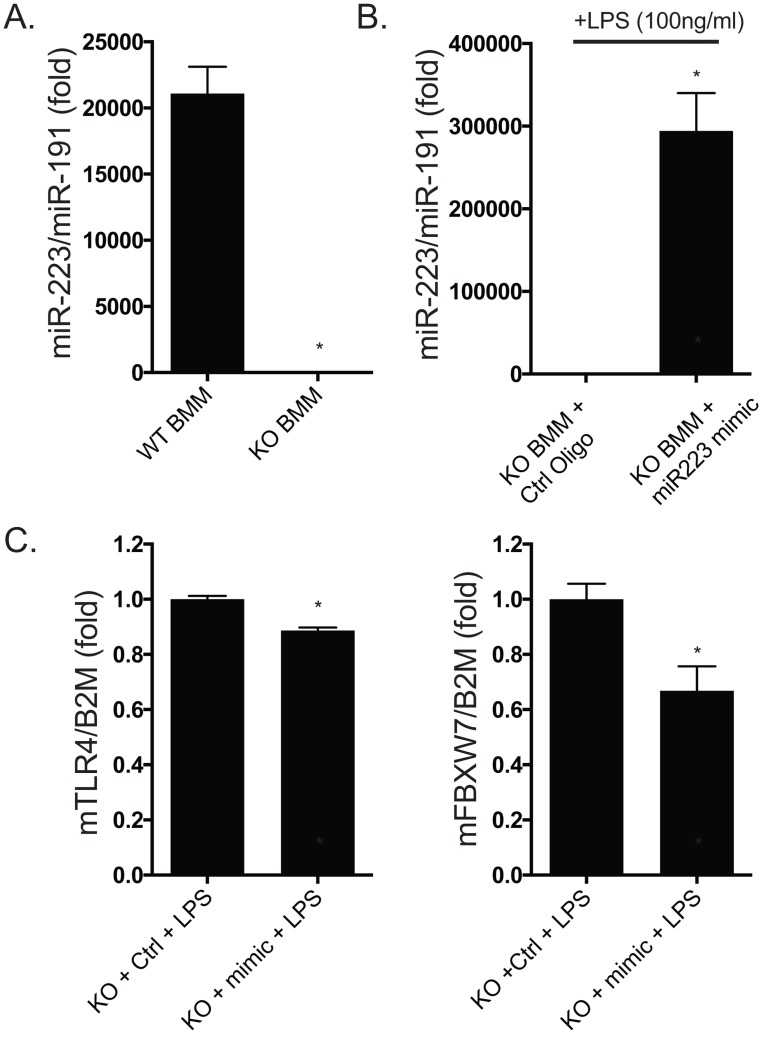
miR-223 mimic reduces TLR4 expression in macrophages stimulated with LPS. (A) WT BMM have about 21,000-fold greater expression of miR-223 by TaqMan probe-based qPCR compared to miR223KO BMMs. (B) miR223KO BMMs electroporated with miR-223 mimic at 500nM have approximately 290,000-fold higher expression than miR223KO BMMs electroporated in the presence of 500nM of mimic control oligo. (C) qPCR of total cDNA (RNA used in B) shows that the miR-223 mimic (“mimic”) significantly decreases mTLR4 and mFBXW7 (pan-detecting primer) in the presence of LPS, compared to BMM not receiving miR-223 mimic (“Ctrl,” control oligo) relative to beta-2 microglobulin (B2M).

miR223KO BMMs electroporated with miR-223 mimic (500nM) had levels about 10-fold higher than WT BMMs (Fig 6A and 6B). Optimizing transfection in primary macrophages is challenging and our goal was to replete the miR223KO BMMs to a level reasonably comparable to WT (within a physiologic range). We believe we were successful in this regard. Further, miR22KO BMMs receiving miR-223 mimic had significantly decreased RNA expression of TLR4 and FBXW7 (pan-detecting primer) in the presence of LPS, compared to BMMs not receiving miR-223 mimic, as measured by qPCR (Fig 6C).

## Discussion

In this study, we show that miR-223 was the most dramatically altered miRNA species measured in the VAT of obese and insulin resistant humans. Further, we showed that miR-223 upregulation in the VAT was specific to the SVF. The SVF contains largely VAT leukocytes, but also contains adipocyte progenitor cells and endothelial cells. However, we believe that the miR-223 levels we report are largely due to the VAT macrophage population because it has been clearly shown that miR-223 is most abundantly expressed in myeloid-derived cells, though other cell and tissue types do express miR-223 at lower levels [13, 14]. Peripheral human blood monocytes miR-223 levels were no different between lean and obese groups, supporting our interpretation that the obesity-associated miR-223 induction is specific to changes in tissue macrophage phenotype. Based on these findings and bioinformatics predictions we hypothesized that miR-223 expression in VAT macrophages regulated both pro- and anti-inflammatory pathways including the E3 ubiquitin ligase FBXW7.

As part of the E3 ubiquitin ligase complex, FBXW7 protein functions to target multiple proteins for ubiquitination and subsequent degradation [15]. While the function of FBXW7 has been most thoroughly reported in cancer, FBXW7 has also been clearly shown to downregulate TLR4 (and inflammation) in human and murine macrophages by the Sterneck group [6]. Our data showing miR-223-mediated repression of FBXW7 in macrophages as well as the downregulation of miR-223 upon LPS stimulation strongly support a role for miR-223 in the interplay between FBXW7 and TLR4. We see that removal of miR-223 increases both TLR4 and FBXW7 levels, though unstimulated knockout macrophages have a higher normalized TLR4/FBXW7 ratio, indicating an enhanced ability to respond to LPS. After LPS (or IFN**γ**/LPS) treatment, the difference in the TLR4/FBXW7 ratio was no longer significant between WT and KO. Our data suggests a miR-223-FBXW7-TLR4 regulatory axis, with macrophage miR-223 levels likely modulating the inflammatory response to LPS via effects on FBXW7 and TLR4 expression. The upstream mechanism by which this occurs, however, is unclear.

TLR4 activation has been well-linked to the etiology of insulin resistance in obesity, contributing to meta-flammation most often via ligation by palmitic acid [5, 16], levels of which are increased in obesity [17, 18]. With respect to FBXW7 expression in obesity and insulin resistance, FBXW7 was reported to be significantly lower in the liver of a high fat diet murine model of non-alcoholic fatty liver disease (NAFLD) with a putative link to regulation of hepatic SREBP1 levels [19]. Interestingly, liver specific FBXW7 ablated mice developed NAFLD [20]. Our data, combined with these previous reports, suggest that miR-223 expression changes in the liver may further modulate an FBXW7-SREBP1 connection.

miR-223 is known to play a conserved role in the development and homeostasis of myeloid and granulocyte differentiation. Expression of miR-223 is regulated by several transcription factors, including transcription factor PU.1, CCAAT-enhancer-binding proteins (C/EBP)-α and -β and nuclear factor I-A (NFI-A) [14]. miR-223 regulates the production of nucleotide-binding oligomerization domain-like receptor protein 3 (NLRP3) and IL-1β [21]. A variety of pathogens or cell stressors are capable of inducing NLRs which form the inflammasome complex. NLRP3 has been recently shown to play a key role in the pathogenesis of insulin resistance and obesity [22], and is regulated by compounds such as ceramide, which induce IL-1β processing via caspase-1 activation. Following stimulation by a Toll-like receptor ligand, many cell types require the NLRP3 inflammosome to initiate an inflammatory response and induce IL-1β production. Interestingly, Haneklaus et al. found that overexpression of miR-223 prevented the accumulation of NLRP3 protein and inhibited IL-1β production by the inflammosome. Zhuang et al. showed that miR-223 whole body knockout mice developed worsened insulin resistance with high fat diet feeding and diet-induced obesity [23]. The authors attributed their result to miR-223-mediated suppression of Pknox1 function, a positive regulator of macrophage activation. However, we did not find that Pknox1 expression was altered in human or murine obesity. We believe that the role of miR-223 in meta-flammation is multifactorial and involves regulation of FBXW7, TLR4, STAT signaling, and nitric oxide (NO) production in macrophages.

Ablation of miR-223 resulted in enhanced IFNγ/LPS-induced NOS2 expression. Macrophage-derived NO plays a critical role in the etiology of insulin resistance as a potent source of oxidative stress and protein S-nitrosylation of many cellular components [24]. NO also functions in vasodilation, thus miR-223 expression in obesity may have implication in cardiovascular changes associated with macrophage inflammation (foam cells, atherogenesis) [25]. We believe it is likely that upregulation of miR-223 in human obesity has a net suppressive effect on the inflammatory cascade in visceral adipose tissue macrophages representing a compensatory adaptation to the inflammatory disease. However, our study demonstrates that miR-223 levels potently regulating macrophage FBXW7, a key suppressor of TLR4.

## Supporting Information

S1 File qPCR data. (XLSX)

## Abbreviations

mBMM: mouse bone marrow-derived macrophages
DIO: diet-induced obesity
HFD: high fat diet
SCD: standard chow diet
UTR: untranslated region
VAT: visceral adipose tissue
IR: insulin resistance
